# Cell‐free synthesis of silver nanoparticles in spent media of different *Aspergillus* species

**DOI:** 10.1002/elsc.202200052

**Published:** 2023-01-12

**Authors:** Alexander Boldt, Jan Walter, Fabian Hofbauer, Karen Stetter, Ines Aubel, Martin Bertau, Christof M. Jäger, Thomas Walther

**Affiliations:** ^1^ Institute of Natural Materials Technology TU Dresden Dresden Germany; ^2^ Institute of Chemical Technology TU Freiberg Freiberg Germany; ^3^ Department of Chemical and Environmental Engineering University of Nottingham Nottingham UK; ^4^ Data Science and Modelling, Pharmaceutical Sciences, R&D AstraZeneca Gothenburg Mölndal Sweden

**Keywords:** Aspergillus, nanoparticles, precious metals, reduction, wastewater

## Abstract

The recovery and valorization of metals and rare earth metals from wastewater are of great importance to prevent environmental pollution and recover valuable resources. Certain bacterial and fungal species are capable of removing metal ions from the environment by facilitating their reduction and precipitation. Even though the phenomenon is well documented, little is known about the mechanism. Therefore, we systematically investigated the influence of nitrogen sources, cultivation time, biomass, and protein concentration on silver reduction capacities of cell‐free cultivation media (spent media) of *Aspergillus niger*, *A. terreus*, and *A. oryzae*. The spent medium of *A. niger* showed the highest silver reduction capacities with up to 15 μmol per milliliter spent medium when ammonium was used as the sole N‐source. Silver ion reduction in the spent medium was not driven by enzymes and did not correlate with biomass concentration. Nearly full reduction capacity was reached after 2 days of incubation, long before the cessation of growth and onset of the stationary phase. The size of silver nanoparticles formed in the spent medium of *A. niger* was influenced by the nitrogen source, with silver nanoparticles formed in nitrate or ammonium‐containing medium having an average diameter of 32 and 6 nm, respectively.

**Abbreviations**
AN: Aspergillus niger
AT: Aspergillus terreus
AO: Aspergillus oryzae
MPEC: Microparticle enhanced cultivation
TEM: Transmission electron microscopy

## 1 INTRODUCTION

The recovery and valorization of precious metals and rare earth metals from wastewater are of great importance to prevent environmental pollution and recover valuable resources. Precious metals present in industrial wastewater can be recycled by pyrometallurgical [1, 2], hydrometallurgical [3], and biohydrometallurgical [4] processing, with the latter being the most energy‐ and cost‐efficient strategy of the three. Drawbacks of this technology, however, are long batch treatment times and lower yields, when the used microorganism is growth‐inhibited by the metal ions or other components of the wastewater.

Therefore, alternative methods for the recovery of precious metals from highly cytotoxic waste streams, such as wastewater from the galvanic industry and production residues of platinum‐based anti‐cancer drugs, are currently explored. One such alternative is the addition of cell‐free spent cultivation media to the wastewater rather than treatment with living cells. Components present in the cell‐free media catalyze the reduction of the metal ions and formation of nanoparticles from the colloidal metals, which can then be recovered from the wastewater [5, 6, 7, 8]. This method not only represent a convenient means for recovering metals from wastewater but has the advantage of generating a high‐value product. The nanoparticles that are formed during the process also have interesting physicochemical properties that can be exploited in several ways. Silver nanoparticles, for instance, are needed for many different applications: the production of optical sensors [9], photovoltaics [10, 11], semiconductor devices [12] as well as for producing disinfectants and bactericidal agents in health care and medicine [13, 14, 15]. Therefore, they sell at higher prices than raw metal, which can favorably contribute to the economic viability of metal recovery processes.

Because of such advantageous process characteristics, we chose to investigate nanoparticle formation using cell‐free spent cultivation media; and since they have a particularly broad field of potential applications, we focused on the investigation of the production of silver nanoparticles. Various microorganisms are capable of forming silver nanoparticles intra‐ and extracellularly [16]. However, the nanoparticle production capacities of fungi were most frequently studied. For instance, representatives of *Fusarium* [17, 18, 19, 20], *Cladosporium* [21], *Verticillium* [22], *Penicillium*, and *Aspergillus* [5, 23, 24, 25, 26, 27, 28] were investigated regarding their ability to extracellularly synthesize nanoparticles. Despite the numerous reports on silver nanoparticle production by those fungi, the exact mechanism of nanoparticle formation remains elusive. For instance, the hypothesis of the involvement of nitrate reductase in the reduction reaction has been frequently raised [6, 29, 30]. However, a recent study reported silver ion reduction by the cofactor NADPH without the need of any enzyme and, which challenges the model put first forward by Durán et al. [31]. In addition, the factors that affect nanoparticle formation from the colloidal silver, for example, regarding the size distribution of the particles or their coagulation, are widely unknown.

**PRACTICAL APPLICATION**

Recovery of precious metals from industrial wastewater streams is important to prevent environmental pollution and to save valuable resources. Cell‐free spent cultivation media of different fungi have been shown to reduce silver ions and to form silver nanoparticles. In this work, we developed quantitative methods to monitor this process and to compare nanoparticle formation depending on different *Aspergillus* species and cultivation conditions. Among the tested strains, *A. niger* was identified as the most potent organism to produce silver nanoparticles.

Hence, to optimize silver recovery from wastewaters, further chemical analyses are necessary to identify compounds that are essential for both silver reduction and particle formation. In addition, physiological studies of the fungi are needed to optimize cultivation conditions and increase the production of these compounds, as well as to identify (and possibly modify) the metabolic pathways for their production. In previous work, the identification of active compounds required for silver ion reduction was hampered by the use of complex cultivation media [17, 32, 33], which contain a large spectrum of different organic and inorganic substances and impurities, all of which may influence the process in different ways. Moreover, the application of environmental fungal isolates for silver nanoparticle formation complicates physiological and functional genomics analyses as genomic and detailed biochemical information for these fungi is often missing [34, 35]. Potentially even more important, several of these strains are pathogens [17, 36, 37], which restrict their use in industrial applications.

In this study, we provide the experimental basis for overcoming these problems by using an adapted and well‐defined mineral medium for cultivations and nanoparticle production experiments. In addition, we developed a quantitative method to monitor silver ion reduction capacity and yield, enabling quantitative comparison between fungal species and cultivation conditions. To aid future physiological analyses, we applied these methods to investigate silver nanoparticle formation in spent media of three well‐characterized non‐pathogenic *Aspergillus* strains, that are, *A. niger* ATCC1015*, A. oryzae* RIB40, and *A. terreus* NIH2624. These strains are sequenced [38, 39, 40] and their metabolic capacities are comparatively well described [41, 42, 43, 44, 45], which may help future rational metabolic engineering of these strains to increase their silver ion reduction and nanoparticle formation capacities.

## 2 MATERIALS AND METHODS

### 2.1 Chemicals and reagents

All chemicals and solvents were purchased from Carl Roth or Merck unless otherwise stated.

### 2.2 *Aspergillus* strains

In this study, the fully sequenced *Aspergillus* sp. *Aspergillus niger* ATTC 1015 [38], *Aspergillus terreus* NIH 2624 [39], and *Aspergillus oryzae* RIB40 [40] were used. These strains were provided by Matthias Brock (University of Nottingham, UK).

### 2.3 Media and cultivation conditions

All cultivations were performed at 30°C for 5 days on a rotary shaker (Infors HT, Bottmingen, Switzerland) at 150 rpm in 100 ml shake flasks. The standard cultivation volume was 30 ml. A 15 g/L talc (Mg_3_Si_4_O_10_(OH)_2_) were added to the cultures for ensuring homogeneous pellet formation [46, 47, 48]. For standard propagation and spore cultivation, Medium 90 (M90; DSMZ, Braunschweig, Germany) was used. The mineral media used in this study was based on the widely used M9 mineral medium and consisted of a base medium (0.15 g/L KH_2_PO_4_, 0.15 g/L K_2_HPO_4_, 0.1 g/L MgSO_4_·7 H_2_O, 0.22 g/L calcium lactate·5 H_2_O, 0.005 g/L FeSO_4_·7 H_2_O, 0.005 g/L NaCl, 100 mmol/L HEPES and 10 g/L sucrose). Sucrose was selected as C source based on preliminary tests (Figure S1). The base medium was supplemented with one of the following nitrogen sources: 10 g/L NaNO_3_, 10 g/L NaNO_2_ or 7.5 g/L (NH_4_)_2_SO_4_. The stock solution of trace elements consisted of 0.4 g/L EDTA(Na_2_)·2H_2_O, 1.8 g/L ZnSO_4_·7H_2_O, 1.2 g/L MnSO_4_·H_2_O, 1.8 g/L CoCl_2_·6H_2_O, 1.2 g/L CuSO_4_·2H_2_O, 0.4 g/L NaMoO_4_·2H_2_O, 0.1 g/L H_3_BO_3_, and 0.1 g/L KI dissolved in double‐distilled water and sterile filtered. The pH of all mineral media was determined to be 6.0.

### 2.4 Generation of the spore inoculant

Cell culture flasks (Greiner, Austria, surface area 75 cm^2^, canted neck, with cap, filtered) were prepared with 20 ml of M90 agar and inoculated by streaking 200 μl of fungal cultures growing in M90. After growth for two weeks at 30°C, the spores were harvested by suspending the air mycelia in 25% (v/v) glycerol. The resulting suspensions were adjusted to a final concentration of 10^7^ spores per ml in 25% (v/v) glycerol. The spore suspensions were stored at 4°C until use.

### 2.5 Harvest of the spent medium and cell dry weight determination

Cell‐free medium for nanoparticle synthesis was obtained by cultivating the different *Aspergillus* sp. in an adapted mineral medium supplemented with one of the nitrogen sources specified above. After 3–5 days, the cultivation medium was filtered (Whatman, cellulose, pore size 11 μm) to remove the biomass. The filtrate, in the following denoted “spent medium,” was set to pH 7.0 using 4 mol/L KOH, filter‐sterilized (0.2 μm), and stored at 4°C until further use. To determine the cell dry weight, the retained biomass was transferred onto a sterile petri dish and dried in an oven at 65°C. The drying process was continued until the measured dry weight value remained constant. In the case of cultivations in the M90 complex medium, cultivation was terminated after 3 days. The biomass was then harvested, washed with sterile deionized water, transferred to another sterile shake flask, and overlaid with 30 ml of sterile deionized water. Subsequent leaching of the fungal mycelium was performed for an additional 3 days at 30°C and 150 rpm in a rotary shaker. The leached fungal filtrate was set to pH 7.0 using 4 mol/L KOH if needed, filter‐sterilized (0.2 μm) and stored at 4°C until further use.

### 2.6 Estimation of protein content in spent medium and protein separation

Protein concentration was measured via bicinchoninic acid (BCA) assay (Pierce BCA protein assay kit, Thermo Fisher, Waltham, USA) according to the instructions of the manufacturer's protocol. To achieve separation of proteins based on their molecular weight, 10 ml of spent medium were applied to an Amicon Ultra‐15 tube (Merck, Darmstadt, Germany) with a nominal molecular weight limit of 3 kDa. The spent medium was centrifuged for 10 min at 14,000 × *g* (4°C). Filtrate and retentate were subsequently stored at 4°C until used for further experiments.

### 2.7 Quantification of silver ions in solution

The reduction reaction was initiated by adding 1 mmol/L silver nitrate to the spent medium. The insoluble silver salt formation was minimized by using 10‐ to 20‐fold dilutions of the spent medium. The reaction took place in sealable 1.5 ml polypropylene tubes (Sarstedt, Nümbrecht, Germany). Due to the light sensitivity of the reaction, the samples were transferred to an opaque plastic container and the reaction was carried out at 20°C for 30 min. The concentration of silver ions in an aqueous solution was determined using the NANOCOLOR Silver 3 kit (Macherey‐Nagel, Düren, Germany). The manufacturer's instructions were adapted as follows: In the first step, 50‐fold dilutions of the silver reduction assays were made using sterile deionized water. 103 μl of the base liquid R1 from the supplied test tubes together with 50 μl of reagent R2 were transferred into a 1.5 ml polypropylene tube. Total 500 μl of the sample were added. The solutions were homogenized by inverting the tube three times. Total 50 μl of reagent R3, containing the coloring agent, were added and mixed through inversion. Incubation of 10 min at 20°C followed. Subsequently, the absorption change was measured at a wavelength of 620 nm in a photometer (GENESYS 10S, Thermo Fisher Scientific, Waltham, USA). For quantification, a calibration curve between 0 and 1 mmol/L silver ions were prepared. Deionized water was used in the blank reactions and standards were prepared in fresh media supplemented with respective nitrogen sources. Control experiments using fresh mineral medium were analyzed to determine the formation of insoluble silver salts. The decrease of silver ion concentrations due to non‐specific salt formation in the mineral medium was subtracted from the reduction capacities of the spent media samples.

### 2.8 Spectral analysis of nanoparticle formation

To determine the absorption maxima of the nanoparticles in the spent medium, the wavelengths in the range between 300 and 800 nm were scanned using a spectrophotometer (Infinite 200pro, Tecan Trading AG, Männedorf, Switzerland or GENESYS 150, Thermo Fisher Scientific, Waltham, USA). Water and fresh mineral medium were used as blanks for the measurements.

### 2.9 Characterization of produced silver nanoparticles

Silver nanoparticles were characterized by applying transmission electron microscopy (TEM). Samples were deposited onto Graphene Oxide on holey carbon TEM grids (EM Resolutions Ltd, Sheffield, UK) and analyzed in a JEOL 2100F TEM (JEOL, Tokyo, Japan) operating at 200 kV. Images were acquired with a GATAN Orius CCD camera (GATAN, Pleasanton, USA), EDX data were acquired with an Oxford Instruments XMax80 detector (Oxford Instruments, Abingdon, UK). Samples from *A. niger* culture supernatants (ammonium‐containing mineral medium) and *A. niger*, *A. terreus*, and *A. oryzae* (nitrate‐containing mineral medium) were analyzed.

## 3 RESULTS

In the present study, we set out to quantify the ability of spent cell‐free mineral media from different *Aspergillus* species to form silver nanoparticles. In addition, we wanted to evaluate the impact of different cultivation parameters such as the nitrogen source and incubation time and investigated the correlation between the concentration of secreted proteins and the reduction capacity of the media. We found that there was a surprising lack of proven methods in the literature that quantitatively capture the process of silver ion reduction and nanoparticle formation. Therefore, we developed a method for measuring silver ion reduction and verified nanoparticle formation both spectrophotometrically and by transmission electron microscopy (TEM). We first conducted a series of experiments to improve the reproducibility of the cultivation experiments and to assess and optimize the accuracy of the silver ion measurement methods.

### 3.1 Optimization of cultivation and assay conditions

#### 3.1.1 Morphology of cell pellets can be homogenized by the addition of talc microparticles

**FIGURE 1:**
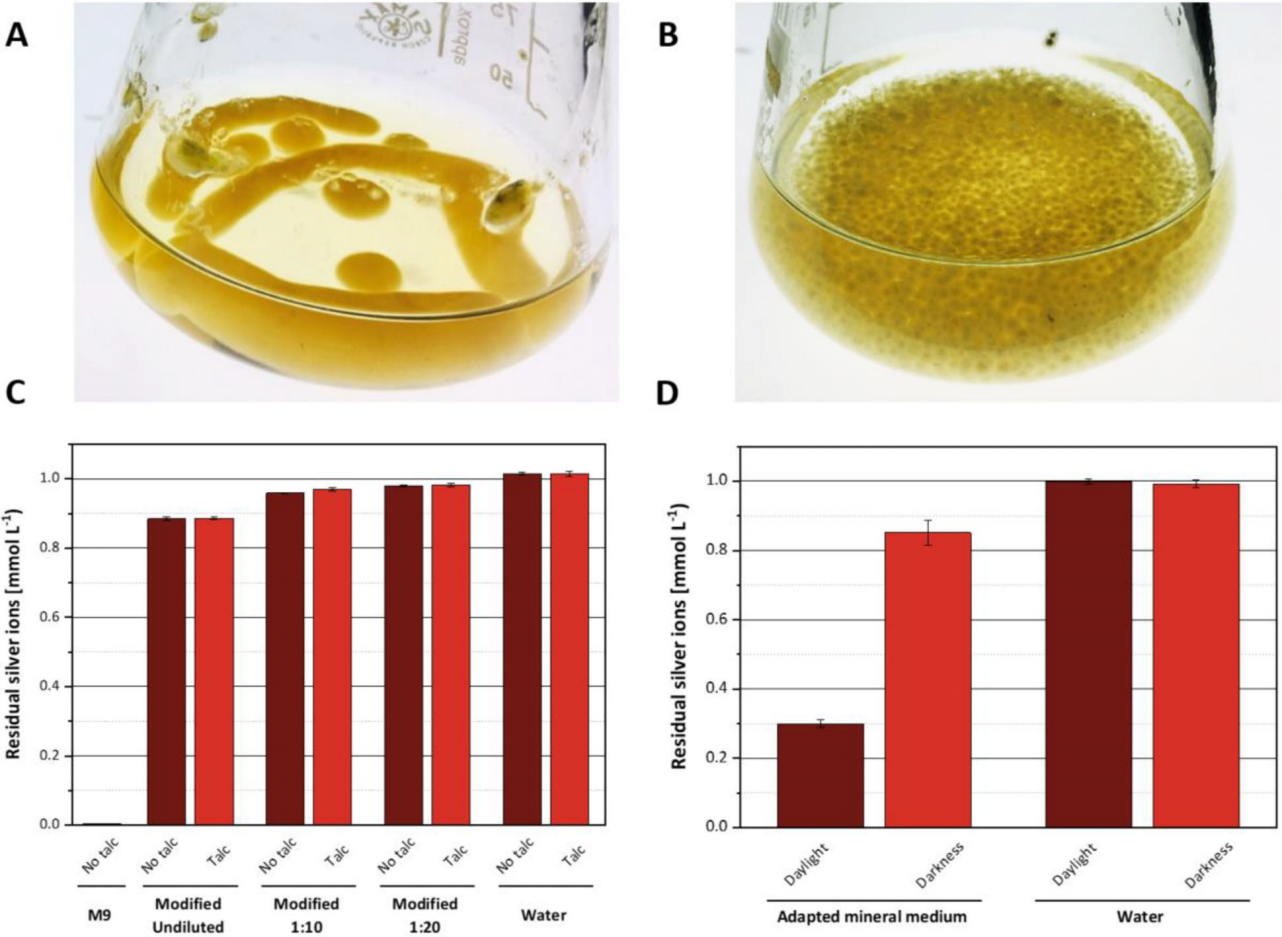
Optimization of cultivation methods and analytical techniques. (**A,B**) Impact of talc supplementation on the morphology of cell aggregates in cultivations of *A. niger* ((**A**) cultivation without talc. (**B**) Culture with 15 g/L talc supplement). (**C**) Residual silver ion concentrations after incubation with the fresh mineral medium. All reactions were carried out using 1 mmol/L silver nitrate under the exclusion of daylight and at 20°C for a period of 30 min. Deionized water was used as a negative control. Error bars represent standard deviation. The medium contained sucrose and ammonium as carbon and nitrogen sources, respectively, and 15 g/L talc. The number of replicates was 3. (**D**) Impact of daylight on residual silver ion concentrations in fresh adapted mineral medium (7.5 g/L ammonia as sole N‐source). All reactions were carried out at 20°C for a period of 30 min under the exclusion of daylight or in bright daylight. Deionized water was used as a control. The medium contained sucrose and ammonium as carbon and nitrogen sources, respectively, and 15 g/L talc. Error bars represent standard deviation from three replicates.

When cultivated in shake flasks, the *Aspergillus* species investigated in this study formed large mycelial pellets of unreproducible morphology (see the morphology of cell aggregates of *A. niger* as an example in Figure 1A). In addition, cell aggregates tended to stick to the glass walls of the flasks, where they were exposed to air and tended to form spores early during the cultivations. Due to these growth morphologies, it was impossible to reliably monitor the cultivation process of the fungi quantitatively and reproducibly. It previously had been reported that the addition of micrometer scale talc particles to submerse cultures of filamentous fungi led to a more homogenous growth morphology, which is characterized by small pellets [48, 49]. This technique is also referred to as microparticle‐enhanced cultivation (MPEC). In agreement with these reports, we found that the addition of 15 g/L talc to the shake flask cultures gave rise to near homogenous pellet morphology and strongly suppressed wall growth and spore formation (Figure 1B).

Based on these positive results, all subsequent growth experiments were performed with the addition of talc to the culture medium.

#### 3.1.2 Optimization of medium composition to minimize the formation of insoluble silver salts

The commonly used protocols for studying silver nanoparticle formation proceed through a cultivation stage, where the fungus is grown on complex media like potato dextrose or malt extract peptone medium (M90), and a leaching stage, where the biomass is first separated from the culture medium and then incubated for several days in pure water [17, 24, 30, 33]. After removing the biomass from this aqueous solution, the sterile supernatant is eventually used to produce nanoparticles. In the present study, we developed a shortened protocol for preparing media suitable for silver nanoparticle formation. Furthermore, we wanted to investigate the impact of the nitrogen source and incubation time, which is tightly related to biomass concentration, on silver ion reduction capacity. Therefore, we decided to use a well‐defined mineral medium for cultivating the fungi and to perform experiments on nanoparticle formation directly in the spent growth medium, thus, avoiding the additional leaching step.

Adjustments had to be made to the composition of the mineral medium to minimize the formation of silver salts. The starting point for media development was the widely used M9 mineral medium [50]. In preliminary tests, the formation of insoluble precipitate was observed immediately after the addition of 1 mmol/L silver nitrate to the M9 medium and no residual silver ions could be detected (Figure 1C). The whitish to gray precipitate dissolved after the addition of 68% (v/v) nitric acid, but was not soluble in water, indicating that the precipitate was indeed composed of silver salts (not shown). Chloride and phosphate anions are known to rapidly form such insoluble silver salts in an aqueous solution. Since both ions are present in the M9 medium at comparatively high concentrations, it was assumed that they were the main source of the unwanted precipitate. Therefore, the content of these ions was strongly reduced. Phosphate ions were reduced by factor 100 to a concentration of 0.3 g/L. Applying this phosphate concentration, approximately 25 g/L biomass can be formed (inferred from a textbook biomass composition of C_1_H_1.8_O_0.5_N_0.2_P_0.03_S_0.02_K_0.008_Mg_0.01_). Since the maximum biomass accumulation in our experiments did not exceed 5 g/L (see below), we concluded that this concentration prevented any phosphate limitation during the cultivations. In addition, we measured phosphate concentrations before and after the addition of 1 mmol/L silver nitrate to the adapted mineral medium and found that they did not change (Figure S2). This result indicated that phosphate concentration in the adapted medium was indeed low enough to prevent the unwanted precipitation of silver phosphate. The concentration of chloride was reduced by replacing NH_4_Cl with the alternative nitrogen sources ammonium sulfate, sodium nitrate, or sodium nitrite, by substituting calcium chloride with calcium lactate, and by supplementing sodium chloride as the sole chloride source at a concentration of 0.005 g/L. All changes to the medium composition are listed in detail in the supporting information (Table S1).

The formation of insoluble silver salts in the modified mineral medium was quantified by adding silver nitrate at a concentration of 1 mmol/L to different dilutions of the medium and measuring the remaining silver ions after 30 min of incubation in the dark. The loss of silver ions due to salt formation could be reduced to approximately 0.12 mmol/L in the adapted mineral medium. When the medium was further diluted (10‐fold to 20‐fold), the formation of silver salts was reduced to approximately 0.02 mmol/L. Based on these results, silver ion reduction in spent media was systematically carried out in 10‐or 20‐fold diluted media. In addition, blank reactions containing fresh mineral medium (in the following “fresh medium” refers to mineral medium never in contact with cells, as opposed to “spent medium” which refers to the cell‐free supernatant recovered after the end of a cultivation) of the corresponding dilution were used to correct measurements of silver reduction for background silver salt precipitation. It is of note that the presence of talc had no impact on the formation of silver‐containing precipitates (Figure 1C).

#### 3.1.3 Shielding silver reduction experiments from light reduces unwanted silver salt precipitation

Preliminary experiments revealed very high variability in measurements of silver ion concentrations in replicate experiments (not shown). This data suggested that small differences in sample handling times caused intolerably high variations of the results. Chemical reactions that implicate silver ions are often strongly impacted by light [51, 52]. Therefore, it was tested whether exposure of the samples to daylight caused significant variations in their silver ion content. To this end, samples of adapted mineral medium containing 1 mmol/L silver nitrate were incubated for 30 min in transparent plastic tubes either in the dark or on the laboratory bench where they were exposed to daylight (Figure 1D). Comparing the remaining silver ion concentrations in both sample types, we found that silver ion content in light‐exposed samples was decreased by 0.7 mmol/L whereas the ion content of samples that were kept in the dark only decreased by 0.15 mmol/L.

From the observation that silver ion concentrations in deionized water did not change in either condition, we conclude that light catalyzed a reaction between components of the mineral medium and the silver ions. While the actual reason for the loss of silver ions remained elusive, these results indicated that all experiments investigating silver reduction and nanoparticle formation should be carried out in the dark to minimize experimental error.

#### 3.1.4 Spectroscopic verification of nanoparticle synthesis in spent growth medium

**FIGURE 2:**
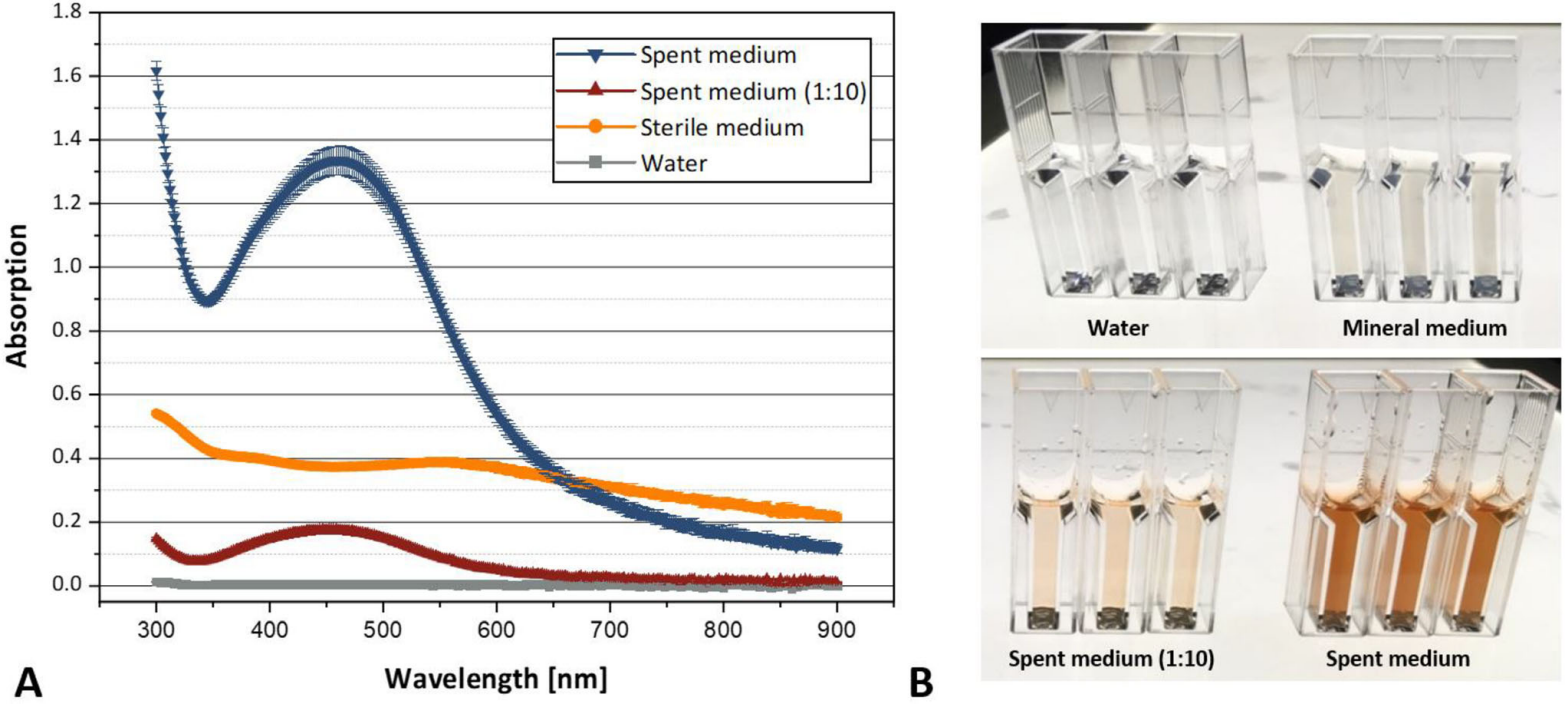
Absorption spectrum of the reduced silver compounds in spent media after 8 h. (**A**) Absorption spectrum of spent medium (*A. niger*/N‐source ammonia), mineral medium (ammonium), and water. Error bars represent standard deviation from three replicates. (**B**) Photographs of the reactions in polystyrene cuvettes (total volume of 1 ml). All reactions were carried out at 20°C for a period of 8 h.

The formation of uncoagulated silver nanoparticles leads to a typical absorption in the range of 450 nm, with small deviations depending on the size of the nanoparticles [53, 54, 55, 56]. Accordingly, we measured the absorbance of different samples in a wavelength scan ranging from 300 to 800 nm. The spent medium of *A. niger* (cultivated in ammonium‐containing mineral medium) was used to check for silver nanoparticle synthesis. Furthermore, undiluted fresh mineral medium and deionized water were used as controls. The incubation time after the addition of 1 mmol/L silver nitrate was extended to 8 h for this experiment to allow not only the initial reduction of silver ions but also the agglomeration and growth of the nanoparticles (see below). The results are shown in Figure 2. Only the spent medium of *A. niger* (both undiluted and 10‐fold diluted) showed a clear absorption maximum in the range of 450 nm. This could also be easily seen visually by a clear red coloration of the reaction mixtures (Figure 2B). While the red‐colored supernatants of the spent media remained completely clear, a whitish precipitate was formed in the control samples containing fresh mineral medium indicating non‐specific salt formation over time. The deionized water sample did not show any precipitation. In summary, these results indicated that silver nanoparticles were indeed formed in the spent growth media of fungal cultivations.

### 3.2 Impact of cultivation parameters on silver ion reduction in spent media

After the successful adaptation of the mineral medium to the requirements for silver ion reduction, we carried out a systematic analysis of the influence of different cultivation parameters on the reduction capacity of spent media from cultures of *A. niger, A. terreus*, and *A. oryzae*.

Previously, it was suggested that nitrate reductase played a key role in silver ion reduction [24, 29, 30, 33]. Since the expression of this enzyme is strongly induced in the presence of nitrate [57, 58], increased reduction efficiency in nitrate‐containing medium could be expected. Apart from the impact on the expression of nitrate reductase as a major factor, large differences in the overall transcriptome have been described in response to different nitrogen sources, suggesting that differences in the panel of secreted metabolites may occur [59]. Therefore, we compared the silver ion reduction capacity of media that contained nitrate to media containing other nitrogen sources such as ammonium or nitrite.

#### 3.2.1 The nitrogen source impacts biomass growth and protein accumulation in spent media

**FIGURE 3:**
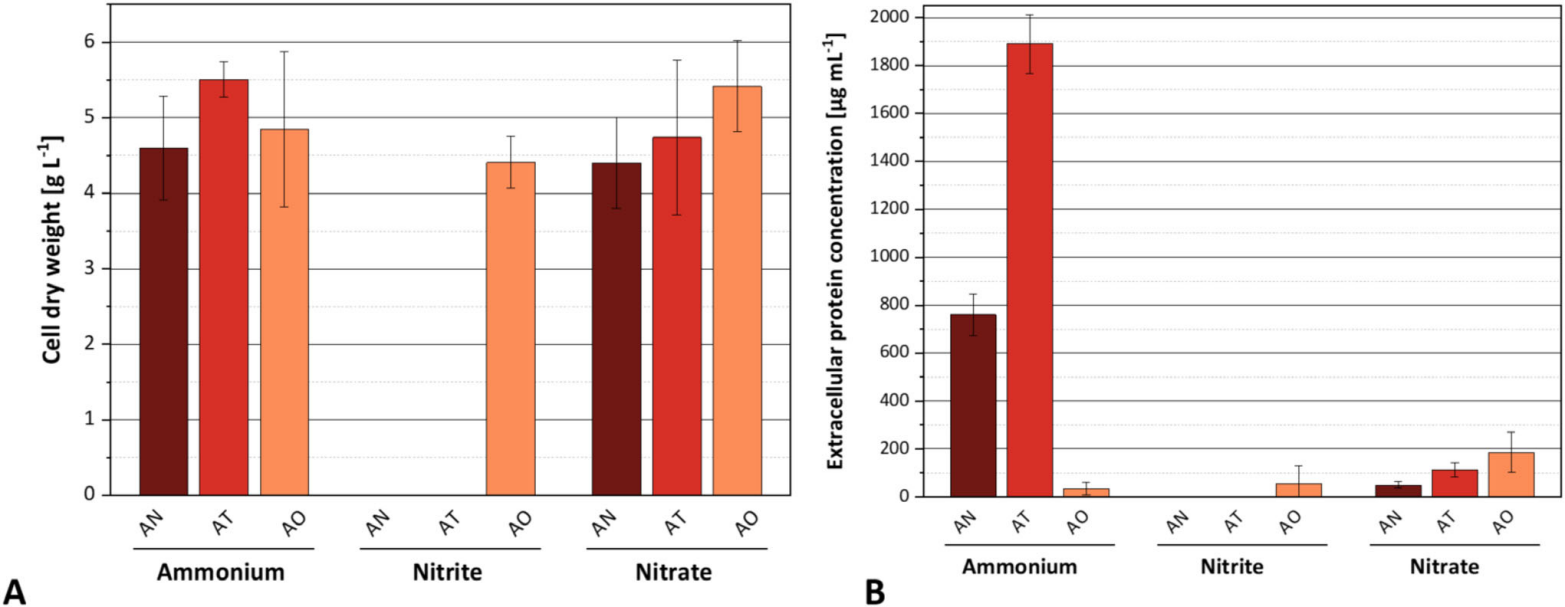
Formation of fungal biomass and determination of the extracellular protein content depending on different nitrogen sources in the mineral medium. AN: *A. niger*, AT: *A. terreus*, AO: *A. oryzae*. (A) Biomass formation and (**B**) extracellular protein concentration after 5 days of cultivation (30°C, 150 rpm; 15 g/L talc). Error bars represent standard deviation from three replicates.

First, the influence of N sources on fungal growth (expressed as cell dry mass) and protein secretion was investigated. The results are shown in Figure 3. After five days of cultivation, biomass accumulation in media containing ammonium or nitrate was comparable, varying between a maximum of 5.5 g/L for *A. terreus* on ammonium and a minimum of 4.4 g/L for *A. niger* on nitrate. Interestingly, *A. oryzae* was the only fungus capable of growing on nitrite under our experimental conditions.

Extracellular protein accumulation varied strongly between fungal species and nitrogen sources (Figure 3B). The highest protein concentrations of 1890 and 761 μg/ml were found in spent media of *A. terreus* and *A. niger*, respectively, when growing on ammonium. Although both fungi accumulated similar biomass concentrations on nitrate, protein secretion was strongly decreased on this nitrogen source reaching only 6.0% and 6.4% of the values obtained on ammonium. *A. oryzae* exhibited only a very small protein secretion on all nitrogen sources that did not exceed 200 μg/ml.

#### 3.2.2 Spent growth medium of *A. niger* has the highest silver ion reduction capacity

In the next step, we investigated which *Aspergillus* species produced the highest silver ion reduction capacity and whether the different cultivation conditions had an influence on the reduction capacity of the spent media. As growth on nitrite could only be observed for *A. oryzae*, we did not further investigate the impact of this nitrogen source but focused on the comparison between ammonium and nitrate. To this end, the silver reduction capacities of the spent media were estimated by adding 1 mmol/L silver nitrate to a dilution series of the spent media and measuring the residual silver concentration after 30 min. Since growth of the fungi on ammonium‐containing medium caused strong acidification of the medium (cultures reached pH ∼2.5 after 5 days), pH of these media was adjusted to 7 by adding potassium hydroxide prior to incubation with silver ions. In the case of nitrate‐containing medium and the experiments with M90 medium, no subsequent adjustment of the pH value was necessary.

**FIGURE 4:**
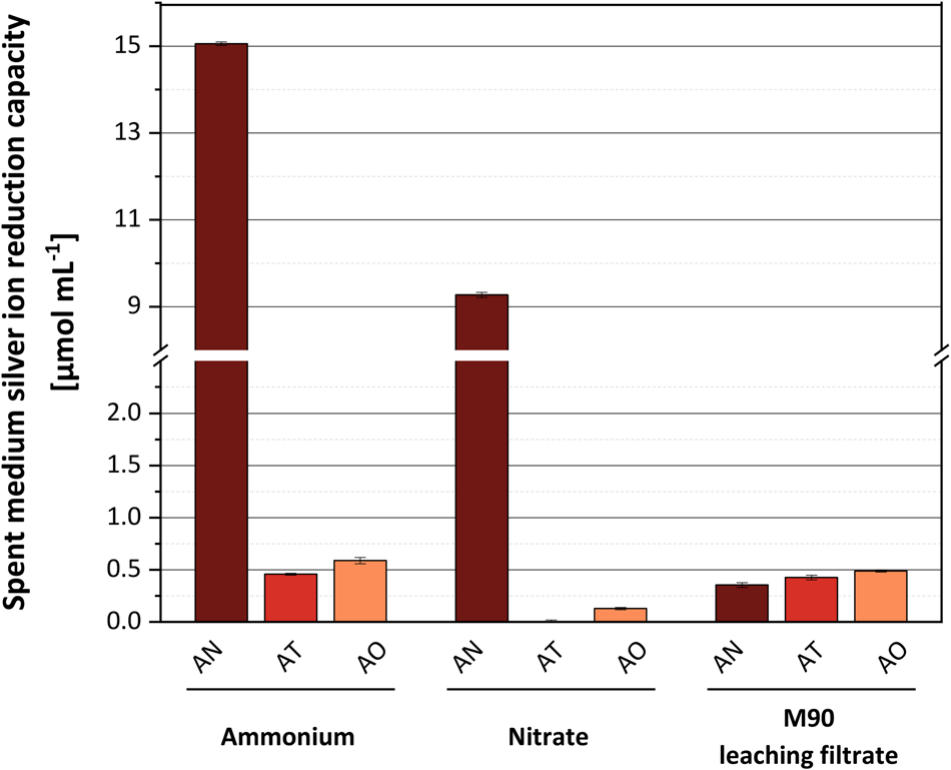
Quantification of silver ion reduction capacity of the fungal spent medium (ammonium or nitrate as sole N source) and leaching filtrate (based on M90 cultivation). All reactions were carried out in a dark environment at room temperature for over 30 min. **AN**: *A. niger*, **AT**: *A. terreus*, **AO**: *A. oryzae*. Error bars represent standard deviation from three replicates.

We found that the spent media of *A. niger* showed the best silver reduction performance on both nitrogen sources (Figure 4). When grown on ammonium, *A. niger* spent medium exhibited the highest reduction capacity of 15 μmol silver ions per milliliter spent medium. This was 33 times higher the reduction capacity obtained for *A. terreus* and 26 times higher than that for *A. oryzae* using the same mineral medium. The second‐highest reduction capacity of 9.3 μmol silver ions per milliliter of the spent medium was also measured for *A. niger* when grown in a nitrate‐containing mineral medium. This was 70 times higher than for *A. oryzae* using the same mineral medium. The nitrate‐containing spent medium from *A. terreus* showed almost no reduction of silver ions.

In addition, we compared the silver ion reduction capacity of spent growth media with the reduction capacity of a medium, which was obtained by a widely used protocol [17, 24, 33, 60]. In this method, the biomass is harvested after cultivation and transferred to pure water, where it is leached for 3 days allowing enzymes and metabolites to accumulate in the supernatant. We found that reduction capacities of the media that were obtained by leaching the biomass of *A. oryzae* and *A. terreus* were in the range of those observed for spent growth media.

In contrast to these results, leaching the biomass of *A. niger* provided a medium that only showed 3.8% and 2.3% of the reduction capacity measured for spent growth media containing nitrate or ammonium, respectively.

#### 3.2.3 Silver ion reduction in the spent medium is not mediated by enzymes

From the results shown above, we could not infer any correlation between silver ion reduction capacity and protein content of the spent media. For instance, the ammonium‐containing spent medium of *A. terreus* had by far the highest protein concentration (1890 μg/ml) but exhibited the lowest reduction capacity of the three fungi. Likewise, *A. niger* secreted 14.5 times less protein on nitrate as compared to ammonium, while the reduction capacity was only reduced by less than a factor of 2. These results suggested that the mechanism of silver ion reduction might be independent of secreted proteins or that the total amount of proteins is not representative of the concentration of an elusive silver reducing enzyme.

Since the ammonium‐containing spent medium of *A. niger* had the highest silver ion reduction capacity, we used this medium to test whether enzymatic reactions were involved in the reduction of the silver ions. To this end, proteins were separated from the spent medium by passing the samples over a filter membrane of a size exclusion limit of 3 kDa. Both the filtrate, which contained no peptides bigger than 3 kDa, and the retentate, which contained all other peptides and proteins secreted by the fungus, were examined concerning the reduction of silver ions and the production of silver nanoparticles.

**FIGURE 5:**
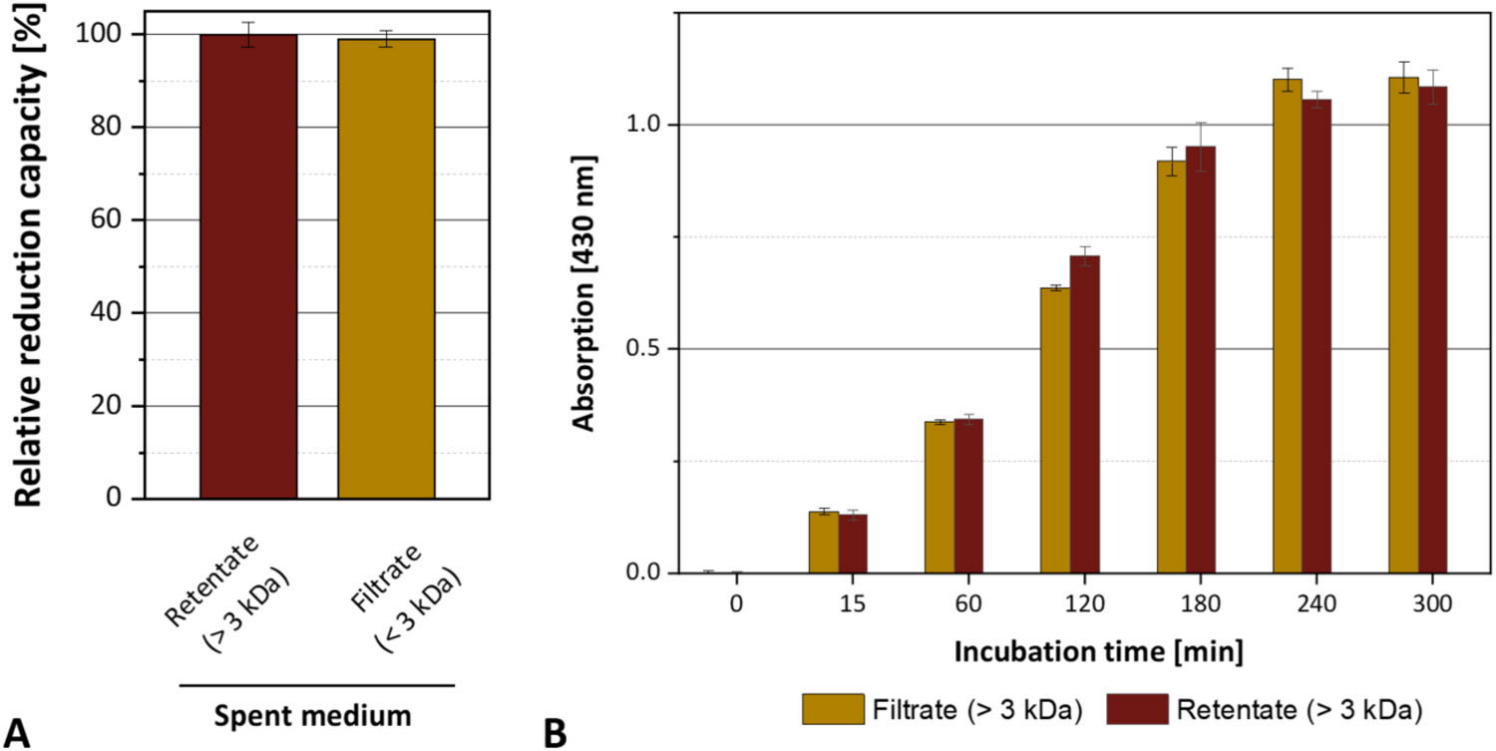
Silver reduction and nanoparticle formation by the filtrate or retentate of the spent growth medium of *A. niger* cultivations. (**A**) Determination of the relative reduction capacity. (**B**) Formation of silver nanoparticles in filtrate and retentate. Absorption at 430 nm indicates the production of uncoagulated silver nanoparticles. The medium contained ammonium as the nitrogen source. All reactions were carried out at room temperature. Error bars represent standard deviation from three replicates.

We found that both the silver reduction and the production of silver nanoparticles occur with the same yield and kinetics in the filtrate and retentate of the spent medium (Figure 5). These results showed that neither of these processes depends on the presence of enzymes or proteins. Notably, our data showed that nitrate reductase, which has a size of 96.7 kDa [58], is not implicated in silver ion reduction, which is at variance with previous reports [24, 29, 30, 33, 61, 62].

#### 3.2.4 The reduction capacity of spent growth medium does not correlate with biomass concentration

**FIGURE 6:**
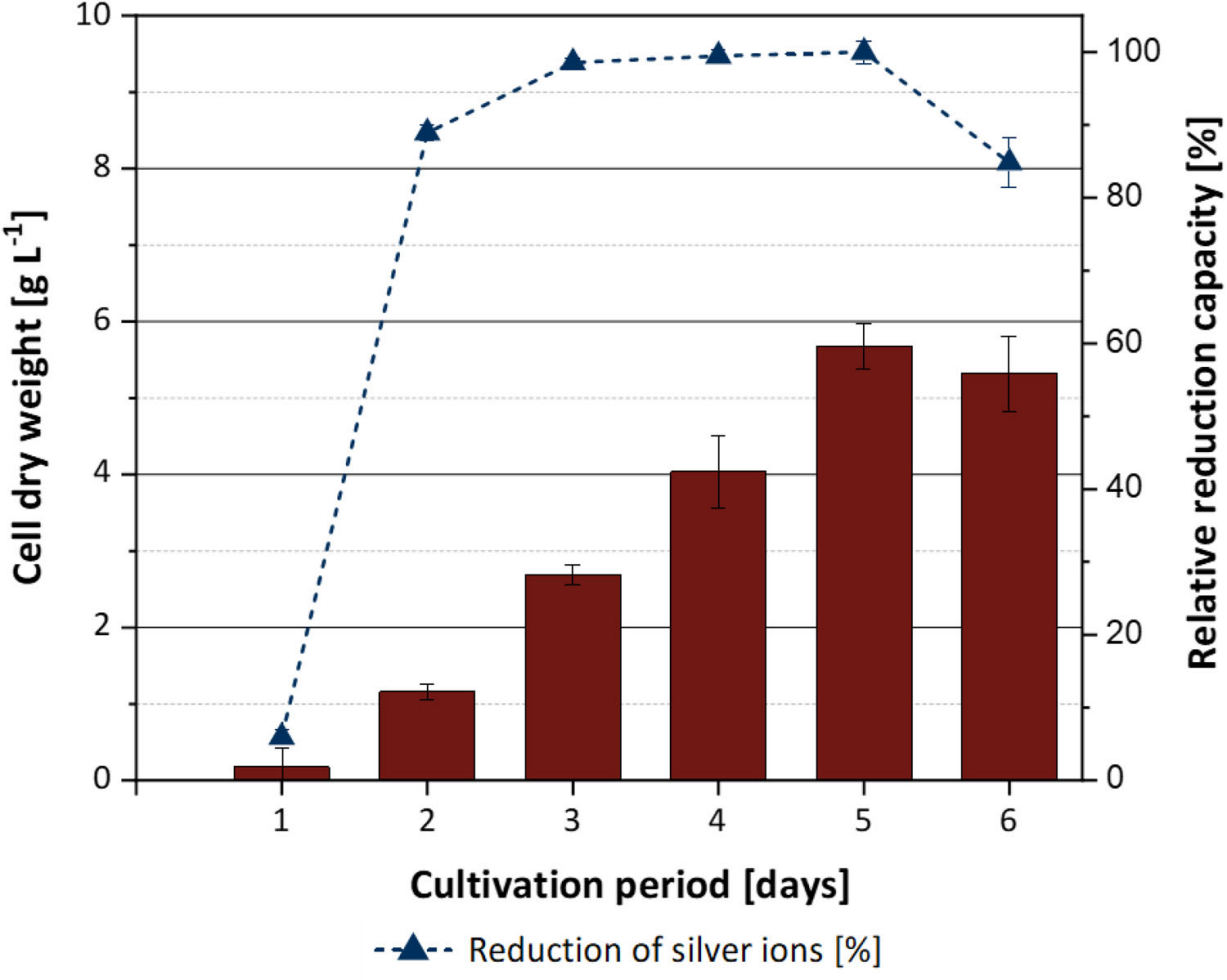
Time course of cell concentration (red bars) in shake flask cultivations of *A. niger*, and relative silver reduction capacity of the spent medium (dashed line). Error bars represent standard deviation from three replicates.

After having established that the silver ion reduction capacity of the spent growth culture media was far higher than the reduction capacity of leaching filtrate, we investigated to which extent the growth phase of the fungal culture at the time of harvest affects the reduction capacity of the spent medium. Since cultures of *A. niger* showed the highest reduction capacity, we conducted these experiments using ammonium‐containing growth media of this organism. The fungus was inoculated into mineral medium and biomass accumulation and silver ion reduction capacity were monitored by taking daily samples over a total cultivation period of 6 days. The results are shown in Figure 6. We observed that a very high reduction capacity of 86% of the maximum value was reached already after 2 days of cultivation. Given that the biomass concentration was only 20% of the maximum value at this time point, it can be concluded that the reduction capacity is not related to biomass concentration.

These results indicated that the time for producing spent medium with high silver ion reduction capacity could be significantly shortened when compared to the conventional leaching protocols. On the other hand, this observation also suggests that the reduction capacity of spent media cannot be increased simply by increasing the biomass concentration of the fungus.

### 3.3 Investigation of silver nanoparticle formation in spent media of *A. niger*

#### 3.3.1 Silver ion reduction and nanoparticle formation occur with a significant temporal delay

**FIGURE 7:**
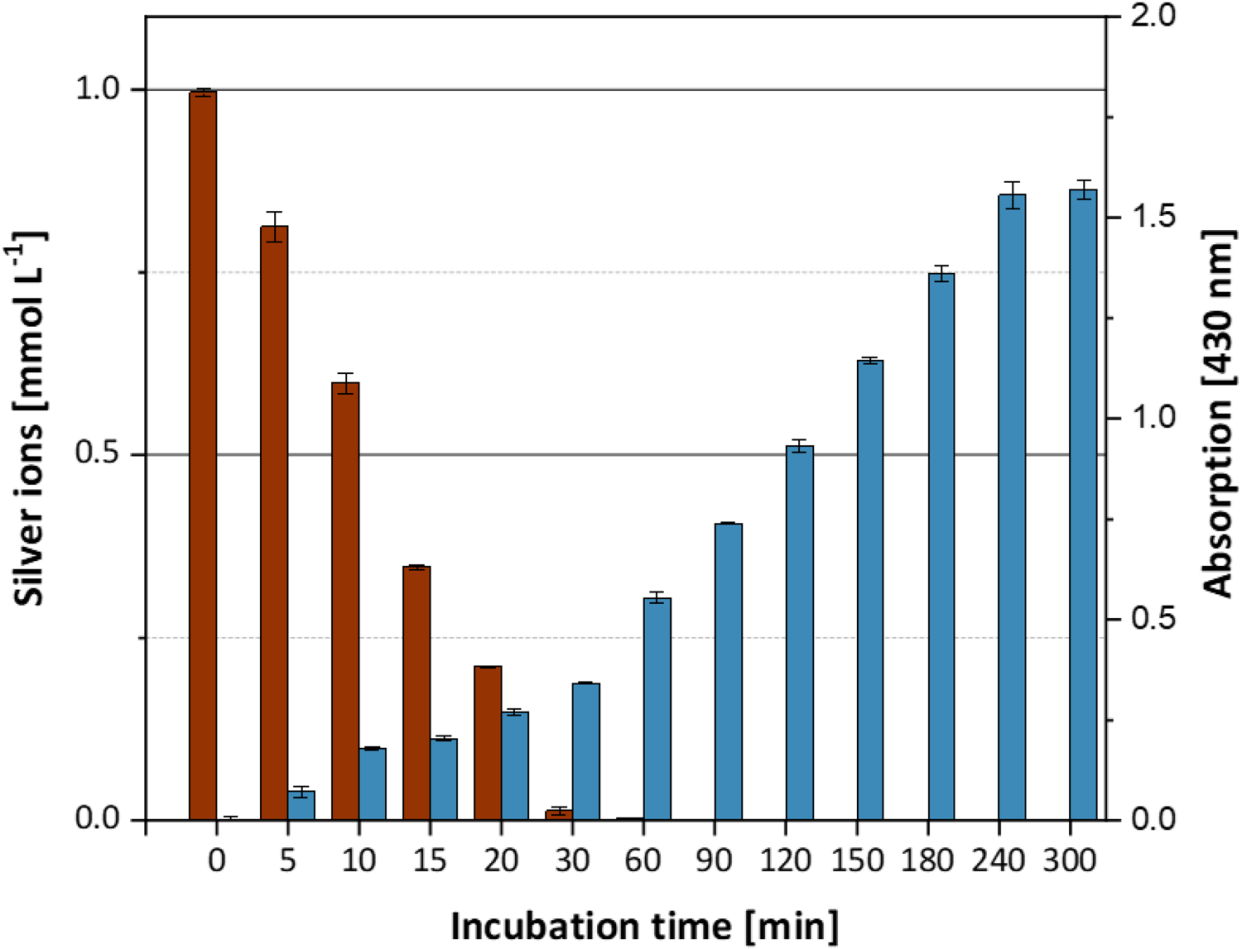
Kinetics of silver reduction and nanoparticle formation in *Aspergillus* spent medium after addition of 1 mmol/L silver nitrate. Red bars: Concentration of silver ions (mmol/L). Blue bars: Absorption at 430 nm indicating the production of uncoagulated silver nanoparticles. The medium contained ammonium as the only nitrogen source. All reactions were carried out at room temperature. Error bars represent standard deviation from three replicates.

We next set out to investigate the kinetics of silver ion reduction and nanoparticle formation. Spent medium from a culture of *A. niger* was incubated with silver nitrate and the time course of both silver ion reduction and nanoparticle formation was monitored. We found that the reduction of silver ions was a comparatively fast process as witnessed by the complete reduction of all ions within the first 30 min of incubation (Figure 5). Nanoparticle production started immediately after elementary silver became available. However, this process was only completed after 4 h of incubation, thus, indicating that nanoparticle formation is much slower when compared to silver ion reduction (Figure 7).

#### 3.3.2 Choice of nitrogen source impacts size distribution of silver nanoparticles

**FIGURE 8:**
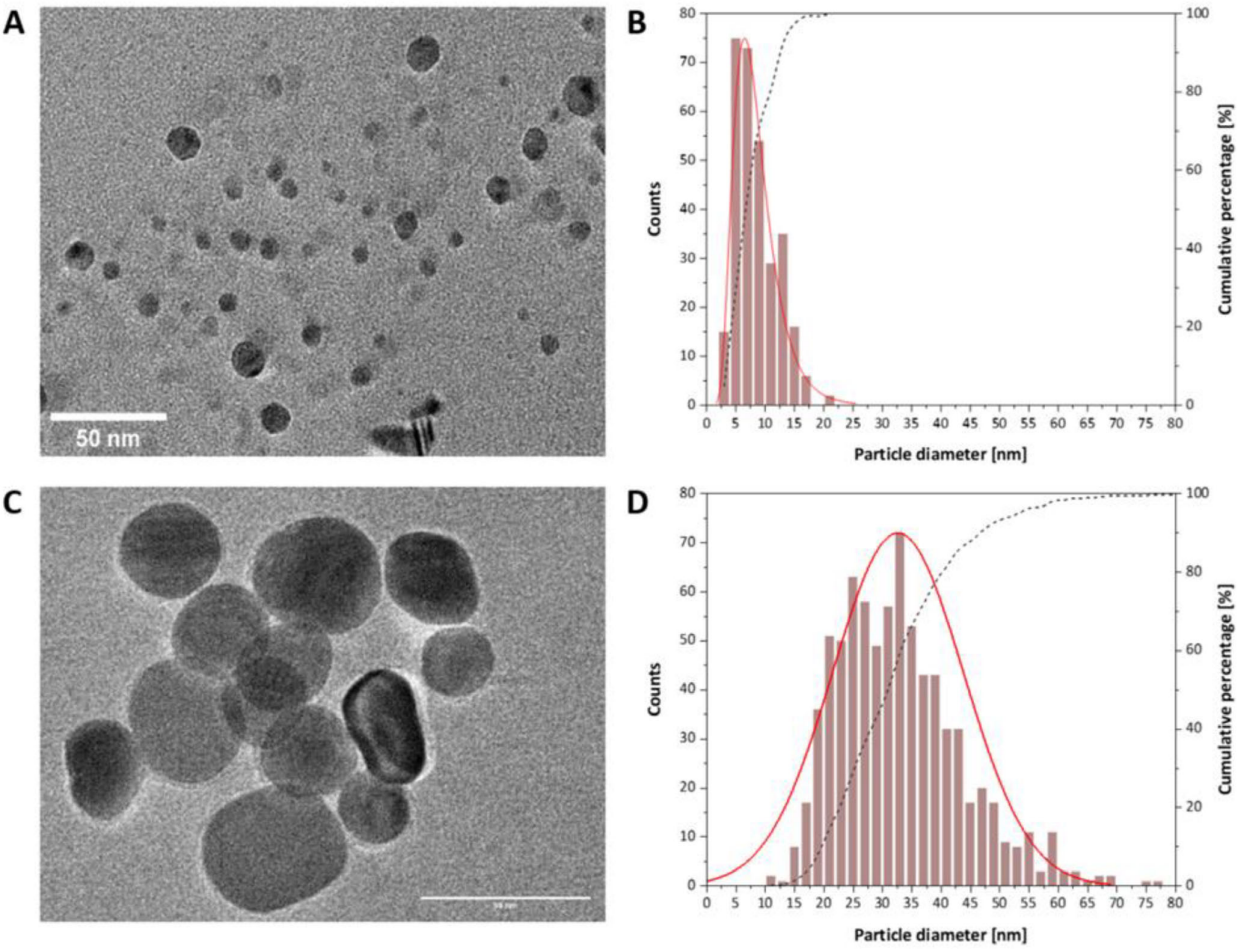
Identification of silver nanoparticles formed in spent medium of *A. niger* cultures using transmission electron microscopy (TEM): Particle size distributions of silver nanoparticles formed in spent media containing ammonium (**A**) or nitrate (C). Electron microscopic images (10^5^ X) of nanoparticles formed in spent media containing ammonium (B) or nitrate (D). The scale bar is 50 nm.

In addition to using spectroscopic analyses, the formation of silver nanoparticles was confirmed by applying Transmission Electron Microscopy (TEM). The presence of nanoparticles could be verified in TEM images of samples taken from ammonium and nitrate containing spent media of *A. niger* (Figure 8). In addition, a detailed analysis of the nanoparticles revealed that their size distribution was strongly impacted by the nitrogen source. When ammonium‐containing spent medium was used for nanoparticle production, the diameter of the nanoparticles varied between 2 and 20 nm having an average size of 6 nm (Figure 8A,B). By contrast, in nitrate‐containing spent medium significantly larger nanoparticles were formed. In this medium, their diameters varied between 10 and 80 nm and had an average size of 32 nm (Figure 8C,D).

To date, only a few studies have been published regarding the influence of media composition on silver nanoparticle formation. It was reported that the choice of the complex medium during the initial growth phase before biomass leaching can influence the nanoparticle formation mediated by the leaching filtrate [33, 36]. We here show that only varying the nitrogen source in an otherwise unaltered mineral medium has a significant impact on the dimensions of nanoparticles.

## 4 DISCUSSION

In this work, we studied the formation of silver nanoparticles in spent media of different *Aspergillus* species. To enable a quantitative comparison between the different fungi and cultivation conditions, we first refined cultivation protocols and analytical methods. In particular, we developed a mineral medium that ensured homogeneous biomass growth (by addition of talc), and which could be directly used for nanoparticle production without an additional leaching stage (by the omission of anions, which form insoluble silver salts). In addition, we measured silver ion reduction using a colorimetric assay. This method gave access to quantitative information on silver ion reduction capacities of different media, which is a strong advantage compared to the commonly used spectroscopic method, which provides only information on the presence of nanoparticles, but not on their actual quantity.

Using these methods, we found that *A. niger* produced spent media with much higher silver ion reduction capacities than the other tested fungi *A. oryzae* and *A. terreus*. Total 1 ml of ammonium or nitrate‐containing spent medium of *A. niger* reduced a maximum of 15 or 9 μmol silver ions, respectively. The reduction capacity of this medium was 30 times higher than that of the medium obtained by leaching the biomass in water. This indicates that commonly used protocols for preparing spent media are not very efficient and that the direct use of spent growth media is preferable when aiming for a high silver ion reduction capacity. Furthermore, we observed that the reduction capacity of the spent medium was not directly related to the biomass concentration at the time of harvest. Specifically, 85% of the maximum reduction capacity was already reached after only 2 days of cultivation, that is, long before maximum biomass concentrations were reached. On the one hand, this observation indicated that protocols for producing spent media for silver ion reduction could be significantly shortened. On the other hand, this result also suggests that the reduction capacity cannot be increased by simply accumulating more biomass. It appears that the fungus secreted silver ion‐reducing compounds at very early stages of growth, which may serve to condition the medium. The identification of these compounds went beyond the scope of this study. However, by observing unimpaired silver ion reduction in spent media after the removal of all proteins, we clearly showed that it was not enzymes, which were responsible for this process. Given that previous works reported the implication of nitrate reductase in silver ion reduction [24, 29, 30, 33, 61, 62], our data shows that the mechanism of silver ion reduction in spent cultivation media has to be revised.

By monitoring silver ion reduction and nanoparticle formation with independent methods, we demonstrated that nanoparticle formation in this biological system occurs with a significant delay compared to the reduction process. This is in line with reports of a two‐step mechanism of silver nanoparticle formation [63]. The reason for this behavior is not clear, but the simplest explanation for this observation is that the formation of three‐dimensional particles requires more time than the reduction of colloidal ions. In any case, monitoring the ion reduction and particle formation separately may provide a means to identify rate‐limiting steps in the overall process that could then be optimized in a more targeted fashion.

Finally, we investigated silver nanoparticle formation in different media using TEM. Interestingly, we found that particle size varied significantly between media containing ammonium or nitrate. It was reported that the size of nanoparticles depends on several factors including fungal species, pH, culture medium, and the presence of capping agents on the nanoparticles [54, 55, 64, 65]. We showed that the use of ammonium as an N‐source gave rise to smaller nanoparticles (6 nm on average) while using nitrate resulted in the formation of larger nanoparticles (32 nm on average). Our study did not elucidate the reasons for the observed size differences. However, tuning the dimensions of silver nanoparticles is relevant for several applications. For example, silver nanoparticles of smaller sizes are highly desired in medical applications due to their enhanced antiviral and bactericidal effect and their reduced level of cytotoxicity to human cells [13, 66]. In contrast, when silver recovery from spent media is the major objective, the utilization of nitrate‐containing growth media is preferable since larger and therefore heavier particles can be separated more conveniently from the liquid.

In summary, our study provides quantitative information on the capacity and kinetics of silver nanoparticle formation in cell‐free spent media of different *Aspergillus* species. In combination with the fact that all investigated fungal strains are sequenced and comparatively well characterized, this provides the basis for future investigations of the biochemical and physiological requirements for nanoparticle formation. Elucidating these requirements will lay the ground for further optimization of the biological recovery of noble and rare earth metals from wastewaters and therefore contribute to a more sustainable use of these resources.

## CONFLICT OF INTEREST

The authors have declared no conflict of interest.

## Supporting information

Supporting Information

## Data Availability

The data that support the findings of this study are available from the corresponding author upon reasonable request.
