# Supplementary material for: Cell‐free synthesis of silver nanoparticles in spent media of different Aspergillus species
Source: Eng Life Sci. 2023 Jan 12;23(3):e202200052. doi: 10.1002/elsc.202200052 (PMC9978913; doi:10.1002/elsc.202200052)
Supplement: Supplementary file 1 — Supporting Information [file ELSC-23-e202200052-s001.DOCX]

# Supplementary information

## Sucrose was chosen as major C-source

To design a suitable mineral medium for the generation of fungal biomass, various carbon sources were tested using *A. oryzae* as a model organism. An important consideration in the selection process was the cost-effectiveness of the mineral medium; accordingly, four inexpensive and commercially available C sources were tested in adapted mineral medium in dependence on 3 different N-sources (ammonium, nitrate, nitrite; 10 g/L each). The standard concentration of C sources was set at 10 g/L. Growth was evaluated based on the settling volume of the mycelium in the shake flask after 5 days of incubation. The results are shown in Figure S-1.

|  |  | **Nitrogen source** | | |
| --- | --- | --- | --- | --- |
|  |  | **Ammonium** | **Nitrate** | **Nitrite** |
| **Carbon source** | **Glucose** |  |  |  |
|  | **Sucrose** |  |  |  |
|  | **Lactose** |  |  |  |
|  | **Acetate** |  |  |  |

**Figure S-1: Growth of A. oryzae in different combinations of carbon and nitrogen sources.** Green color indicates good growth; yellow indicates weak growth and red color indicates no growth of the fungi. Cultivation time: 5 days, 30 °C, 150 rpm.

We could see no growth with lactose and very weak to no growth with acetate as a carbon source. Sucrose and glucose gave comparable biomass formation and huge mycelial pellets were formed using all nitrogen sources. The choice of C source finally fell on sucrose since sucrose is a very cheap C-source and a non-reducing sugar. This will prevent unspecific reduction of the silver ions.

## Determination of phosphate ions before and after silver addition

To verify the formation of silver phosphate in the undiluted adapted mineral medium, the phosphate content was checked by photometric assay before and after the addition of silver nitrate (1 mmol/L) under standard conditions (30 minutes incubation time, exclusion of light, 20 °C). The results are shown in Figure S-2. Since no change in phosphate concentration could be detected, it can be assumed that no or only extremely low formation of silver phosphate occurs under the conditions applied.

**Figure S-2:** Quantification of phosphate ions in the undiluted fresh mineral medium before and after the addition of 1 mmol/L silver nitrate. Error bars represent standard deviation. The medium contained sucrose and ammonium as carbon and nitrogen sources, respectively. The number of replicates was 4.

## Adaptations of the mineral medium starting from M9

The following table S-1 shows the changes with respect to the composition of the mineral media, starting from M9.

**Table S-1: Composition of both M9 mineral medium and adapted mineral medium**

| **M9 mineral medium** | | **Adapted mineral medium** | |
| --- | --- | --- | --- |
| Compound | Concentration (g/L) | Compound | Concentration (g/L) |
| Potassium dihydrogen phosphate | 3 | Potassium dihydrogen phosphate | 0.15 |
| Di-sodium hydrogen phosphate | 6 | Di-Potassium hydrogen phosphate | 0.15 |
| Magnesium sulfate | 0.12 | Magnesium sulfate * 7 H_2_O | 0.1 |
| Calcium chloride | 0.011 | Calcium lactate * 5 H_2_O | 0.22 |
| Thiamine-HCl * 2 H_2_O | 0.35 | Iron(II) sulfate * 7 H_2_O | 0.005 |
| Sodium chloride | 0.5 | Sodium chloride | 0.005 |
| Glucose | 2 | Sucrose | 10 |
| Ammonium chloride | 1 | Respective N-source | 7.5-10 |
